# Anesthetic management of a child with Apert syndrome

**DOI:** 10.4103/1658-354X.76483

**Published:** 2011

**Authors:** Yavor Metodiev, Nadezhda Gavrilova, Atanas Katzarov

**Affiliations:** *Pediatric Anesthesia and Intensive Care, National Trauma Centre “N.I.Pirogov”, Bulgaria*; 1 *Pediatric Anesthesia and Intensive Care, National Trauma Centre “N.I.Pirogov”, Bulgaria*

**Keywords:** *Apert syndrome*, *axillary block*, *transversus abdominis plane block*, *ultrasound guidance*

## Abstract

In this paper, the authors describe an anesthetic technique for a child with Apert syndrome, presenting to the operating room for a syndactyly separation. The anesthetic approach is innovative for the clinic and is a combination of intravenous anesthesia and two regional techniques (axillary block and transversus abdominis plane block, respectively). They were performed under ultrasound guidance and provided analgesia in the two body regions, which were to be operated.

## INTRODUCTION

Apert syndrome includes several abnormalities, such as craniofacial hypoplasia, craniosynosthosis and syndactyly of the hands and feet. In patients, with this syndrome, who come to the operation theatre for craniofacial or extremity surgery, a number of coexisting conditions should be taken under consideration.

## CASE REPORT

A 27-months old girl with Apert syndrome was admitted to the clinic for scheduled syndactyly separation. A written consent form the girl’s mother to publish the case was obtained.

**Figure 1:**
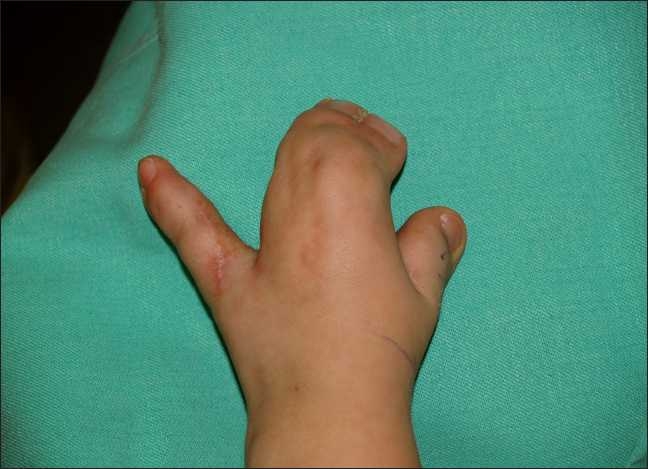
Syndactyly, typical for Apert syndrome

All the components of the syndrome were evaluated preoperatively brachycephaly, multiple syndactylies of the four extremities [Figure 1], orbital hyperthelorism, median facial hypoplasia, mild exophthalmus, and mental retardation. The child underwent an operation for the coronary synosthosis at the age of 9 months. From the medical record and the CT scan, hydrocephaly and atrophy of the cerebral hemispheres were established. Any coexisting malformations of the heart, digestive system, or urogenital system were excluded by medical history and previous medical records. The standard kit of blood tests showed normal results.

After an informed consent, the child was scheduled for an elective surgery under general and regional anesthesia.

The patient was given oral premedication Midazolam 8 mg (0.5 mg/kg) 60 min before anesthesia. An intravenous 22G cannula was inserted in theatre. General anesthesia was induced with 50 mg propofol (3 mg/kg) and then a laryngeal mask ProSeal ^®^ №2 was inserted. The spontaneous breathing of the child was maintained and she was left to breathe 100% oxygen. Anesthesia was maintained with Propofol at a rate of 6 mg/kg/h.

**Figure 2:**
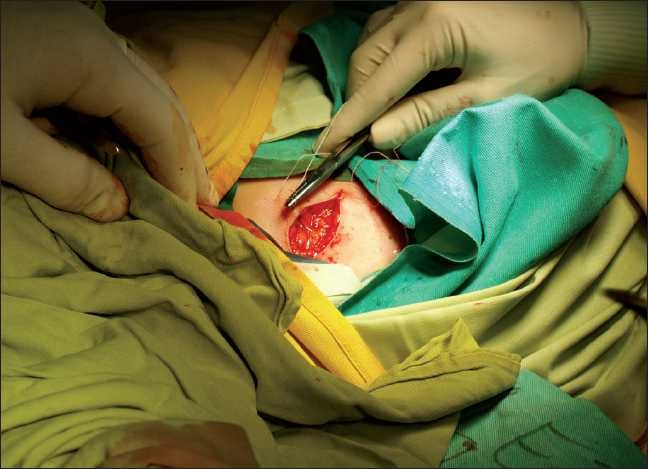
Taking extra skin from the abdominal wall

Besides the routine monitoring (heart rate, respiratory rate, saturation with peripheral plethismogram, and blood pressure), blood glucose, cell blood count, and blood gases were monitored as well. After evaluation of vital parameters, the axillary and transversus abdominis plane (TAP) block were to be performed. The latter was indicated because the surgeon considered the possibility of taking extra skin from the abdominal wall [Figure 2].

Both regional blocks were performed under ultrasound guidance. The equipment consisted of: sonograph SONEO^®^ (Kontron Medical™), linear transducer with frequency 5–10 MHz, two insulated needles STIMUPLEX ^®^A 22G–25 mm (Bbraun™), sterile transparent bandages TEGADERM^®^ 10 × 12 cm (3M™), saline and antiseptic solution. For the axillary block, a solution of 2 ml 0.5% levobupivacaine and 6 ml 1% lidocaine was used, whereas for the TAP block 8 ml 0.25% levobupivacaine.

First, the axillary block was performed. Ultrasound examination of the axillary region was done and the axillary artery and vein were identified, as well as the radial and ulnar nerve. The median nerve was not visualized with certainty. After antiseptic preparation of the axillary region and preparation of transducer, an in-plane technique of axillary block was commenced. The radial and ulnar nerves were approached under constant visualization of the tip of the needle and around each of them 3 ml local anesthetic were injected. The 2 ml left were injected at the spot with the highest possibility of finding the median nerve, according to Retzl *et al*.[1] The spread of local anesthetic around the nerves was obtained.

After the axillary block, TAP block was done. An ultrasound examination of the abdominal wall at the level of midaxillary line was done. The external and internal oblique muscles and the transverse muscle were visualized.

This block was done by in-plane technique. After confirming the correct position of the needle tip by hydrolocation, 8 ml 0.25% levobupivacaine were injected in the transverse fascia plane.

The overall dose of local anesthetics was 3.75 mg/kg for lidocaine and 1.8 mg/kg for levobupivacaine. A routine monitoring was attached during the performance of regional blocks.

A criterion of inadequate analgesia was set by the anesthesia team increase of heart rate and mean arterial pressure by more than 15% than the initial rates. Monitoring rates were registered every 10 min.

As the surgery was advancing more than expected, the risk of regurgitation was increasing. One hundred fifty minutes after the start of surgery a tracheal intubation was conducted and a stomach tube inserted. The surgery lasted for 5 h. An armpit tourniquet was inflated and every 2 h of inflation were followed by 15 min of deflation.

The anesthesia was maintained only with propofol at the rate of 6–8 mg/kg for 200 min. At that time the need for supplementary analgesia in accordance with our criteria was assessed and intravenous fentanyl 1.25 μg/kg was injected four times.

The patient was breathing spontaneously after the tracheal intubation as she tolerated the tube.

Taking the skin graft from the abdominal wall was not followed by any sign of pain.

The patient emerged uneventfully without any signs of respiratory depression or feeling any pain, which allowed an early extubation. Pain was evaluated immediately after surgery according to visual analogue scale and it was zero points. The postoperative follow-ups during the next 24 h did not show the need of opioids because the maximum pain score was three points. No complications, related to anesthesia, were encountered.

## DISCUSSION

Patients with malformative syndromes, like Apert syndrome, sometimes present a great challenge to the anesthesiologist. The most frequently occurring problem is maintaining patient airways.[2] That is why an intravenous induction was preferred rather than an inhalational one. The possibility of impaired ventilation via facial mask because of the facial hypoplasia was predicted. We managed to insert easily the laryngeal mask. Performing the endotracheal intubation later during the surgery was not the best scenario possible, but it was conducted uneventfully. Apert syndrome is in the risk group of regurgitation and aspiration of gastric content.[2] Leaving the laryngeal mask for more than 2 h was another factor that could increase the risk even more. These were the reasons to perform the endotracheal intubation and drain the stomach by inserting a gastric tube. The nasal route for inserting the gastric tube was impassable, probably because of the midline facial hypoplasia.

Preserving the patient’s spontaneous breathing without affecting the quality of analgesia, was our main goal. Here, the advantages of regional techniques for perioperative analgesia were used. Regional anesthesia provides excellent conditions of work to surgeons and reduces perioperative opioid requirements. Thus, it reduces the incidence of opioid side effects, like deep sedation, respiratory depression, nausea and vomiting, itching, etc. In trained hands ultrasound-guided peripheral blocks improve success rate and quality and increase the duration of sensory blockade.[3,4] Besides that, ultrasound guidance decreases the risk of complications, such as vessel puncture and direct neural trauma. Prolonged sensory blockade can be achieved with smaller volume of local anesthetic when using ultrasound guidance.[5] All this allowed us to perform both blocks with reduced volume of local anesthetic without reaching toxic doses and at the same time we could predict an adequate analgesia.

Immediately after emergence and in the early postoperative period the patient did not complain of any pain. This suggested that the need of additional analgesia had not been due to attenuation or inadequacy of sensory blockade. Our conclusion was that the increase in heart rate and mean arterial pressure was caused by “tourniquet pain.”[6] Although the tourniquet was deflated twice, the total time of impaired blood flow of the arm could be the reason for these hemodynamic changes.

## CONCLUSION

We assume that we achieved an optimal anesthetic management of a patient with Apert syndrome. Though, we are aware of the fact that future experience with similar patients can prove us wrong.
